# Thioflavin T as an amyloid dye: fibril quantification, optimal concentration and effect on aggregation

**DOI:** 10.1098/rsos.160696

**Published:** 2017-01-04

**Authors:** Christine Xue, Tiffany Yuwen Lin, Dennis Chang, Zhefeng Guo

**Affiliations:** Department of Neurology, Brain Research Institute, Molecular Biology Institute, University of California, Los Angeles, CA 90095, USA

**Keywords:** Alzheimer's disease, Aβ, yeast prion, fibrillization kinetics

## Abstract

Formation of amyloid fibrils underlies a wide range of human disorders, including Alzheimer's and prion diseases. The amyloid fibrils can be readily detected thanks to thioflavin T (ThT), a small molecule that gives strong fluorescence upon binding to amyloids. Using the amyloid fibrils of Aβ40 and Aβ42 involved in Alzheimer's disease, and of yeast prion protein Ure2, here we study three aspects of ThT binding to amyloids: quantification of amyloid fibrils using ThT, the optimal ThT concentration for monitoring amyloid formation and the effect of ThT on aggregation kinetics. We show that ThT fluorescence correlates linearly with amyloid concentration over ThT concentrations ranging from 0.2 to 500 µM. At a given amyloid concentration, the plot of ThT fluorescence versus ThT concentration exhibits a bell-shaped curve. The maximal fluorescence signal depends mostly on the total ThT concentration, rather than amyloid to ThT ratio. For the three proteins investigated, the maximal fluorescence is observed at ThT concentrations of 20–50 µM. Aggregation kinetics experiments in the presence of different ThT concentrations show that ThT has little effect on aggregation at concentrations of 20 µM or lower. ThT at concentrations of 50 µM or more could affect the shape of the aggregation curves, but this effect is protein-dependent and not universal.

## 1. Introduction

Protein aggregation to form amyloid fibrils is a common feature underlying a wide range of human disorders, such as Alzheimer's disease, Parkinson's disease and type 2 diabetes [1]. Thioflavin T (ThT) is a commonly used probe to monitor *in vitro* amyloid fibril formation. Upon binding to amyloid fibrils, ThT gives a strong fluorescence signal at approximately 482 nm when excited at 450 nm [2]. The mechanism of fluorescence enhancement upon binding to amyloid has been attributed to the rotational immobilization of the central C–C bond connecting the benzothiazole and aniline rings [3–5]. It is commonly accepted that ThT binds to the side chain channels along the long axis of amyloid fibrils [6]. The minimal binding site on the fibril surface has been suggested to span four consecutive β-strands [7,8]. Numerous other studies have been performed to investigate the physico-chemical and spectroscopic properties of ThT upon binding to amyloids fibrils, and the binding mechanism (reviewed in [9–11]). However, several practical questions regarding the use of ThT are still not fully addressed. In this work, we investigate three of these questions: (1) Can we use ThT to quantify the amount of amyloid fibrils? (2) What is the optimal concentration of ThT for monitoring protein aggregation? (3) Does ThT affect the process of protein aggregation?

It has long been recognized that ThT can be used to quantify amyloid fibrils [12]. However, ThT has not been routinely used for amyloid quantification in the literature. Part of the reason may be that there are few systematic studies on the relationship between ThT fluorescence and amyloid concentration to show how robust this method of amyloid quantification is. Identifying the range of ThT concentrations that can be used for amyloid quantification, as well as the range of amyloid concentrations that can be consequently determined, is integral to the usefulness of this method.

Currently, there also lacks consensus on the optimal ThT concentrations for detecting amyloid formation. We identified a wide range of ThT concentrations in the literature, from as high as 64 µM [13] to as low as 1 µM [14], with 20 µM being a popular choice [15–19]. Rationales for choosing such concentrations are rarely discussed. A systematic investigation of ThT binding using a wide range of both ThT and amyloid concentrations would help determine whether there exists an optimal concentration for detection and quantification of amyloid fibrils.

Owing to the wide use of ThT in amyloid detection, it is critical to know whether ThT affects fibrillization kinetics. It is generally assumed that ThT does not significantly affect the kinetics of amyloid formation, but experimental data directly addressing this question are rather limited. Nielson *et al*. [20] reported that ThT did not affect the kinetics of insulin fibril formation at 60°C, but Manno *et al*. [21] suggest that ThT caused a delay in insulin aggregation at 50°C. Foderà *et al*. [22] note that ‘increasing the ThT concentration results in a slightly slower fibrillation process’ in the studies of insulin fibril formation. Xue *et al*. [23] compared β2-microglobulin fibrillization in the presence and absence of ThT and concluded that ThT has no significant effects on β2-microglobulin aggregation. D'Amico *et al*. [24] suggest that ThT promotes Aβ40 aggregation based on their Aβ40 aggregation experiments.

To address these questions outlined above, we have studied the fluorescence of ThT across a wide range of concentrations, from 0.2 to 500 µM, in the presence of three amyloid proteins, Aβ40 and Aβ42 involved in Alzheimer's disease [25], and yeast prion protein Ure2 [26]. We found that the ThT fluorescence intensity correlates linearly with amyloid concentration over a wide range of investigated ThT concentrations. Therefore, ThT can be reliably used as a quantitative probe to measure amyloid concentration. We also show that approximately 20–50 µM ThT gives the highest fluorescence intensity. ThT concentrations at or above 50 µM may change the process of amyloid formation, but this effect is protein-dependent. In general, we recommend 10–20 µM ThT in fibrillization kinetics studies, and 50 µM ThT for the quantification of pre-formed amyloid fibrils.

## 2. Results and discussion

### 2.1. Thioflavin T becomes self-fluorescent at concentrations above 5 µM

**Figure 1.**
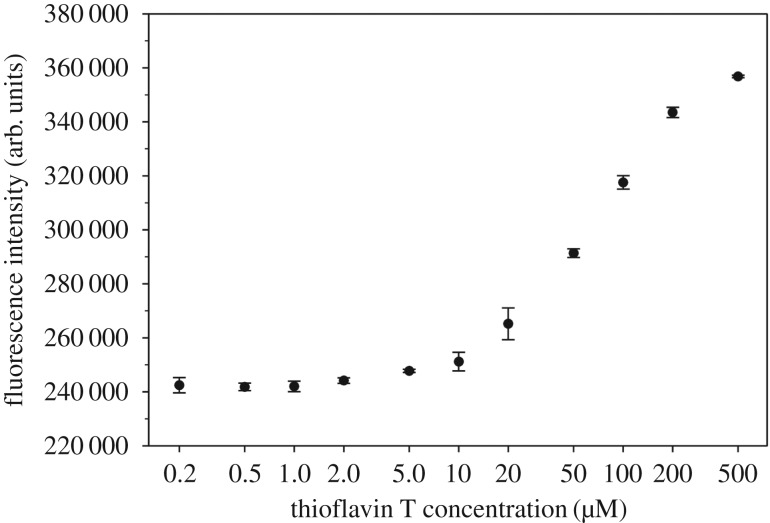
Fluorescence of thioflavin T at various concentrations. Note that thioflavin T becomes self-fluorescent at concentrations of approximately 5 µM or higher. And the fluorescence approaches plateau at 500 µM. Error bars are standard deviations of three independent preparations of thioflavin T.

We first examined the fluorescence of ThT at 490 nm over a wide range of concentrations: from 0.2 to 500 µM. The ThT concentration was determined using absorbance at 412 nm [27,28]. We found that ThT becomes self-fluorescent at concentrations of 5 µM or more in PBS buffer (figure 1). The fluorescence intensity approaches plateau at 500 µM. At 500 µM ThT, the fluorescence intensity is approximately 50% more than background signal from buffer alone. Even at 50 µM, ThT fluorescence signal is 20% more than the baseline level. This suggests that it is important to correct the background ThT signal when higher ThT concentrations are used.

The increase in fluorescence intensity above 5 µM ThT is likely to be due to micelle formation, which has been studied previously by Khurana *et al*. [29]. Khurana *et al*. [29] show that ThT has a critical micelle concentration of approximately 4 µM, in good agreement with our observations that ThT becomes self-fluorescent at or above 5 µM. Formation of ThT micelles was also demonstrated by Sabaté *et al*. [30] and Donnelly *et al*. [31]. To be clear, however, our results do not imply, nor are we suggesting, that ThT binds to amyloid in the form of micelles.

### 2.2. Maximum fluorescence intensity mostly depends on total thioflavin T concentration, rather than amyloid to thioflavin T ratio

**Figure 2.**
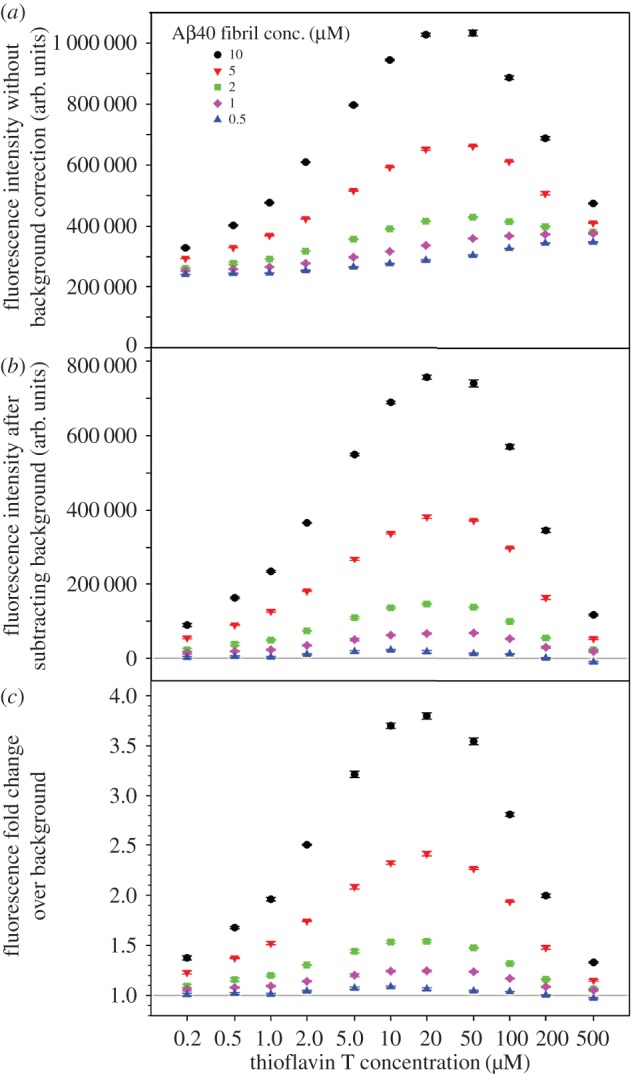
ThT fluorescence at various combinations of Aβ40 fibril and ThT concentrations. (*a*) Raw data without correction of background ThT fluorescence. (*b*) Background ThT fluorescence is subtracted from sample readings. (*c*) Amyloid fluorescence is expressed as fold change over background ThT fluorescence. Error bars are standard deviations of three readings of the same sample and represent instrument measurement errors.

We studied the fluorescence intensity of ThT upon binding to amyloid fibrils at different amyloid and ThT concentrations. The fibrils were sonicated to maximize the number of accessible ThT binding sites on the amyloid. For the amyloid fibrils of Aβ40, we studied a combination of 11 ThT concentrations (0.2–500 µM) and 5 Aβ40 concentrations (0.5–10 µM). As shown in figure 2*a*, when background ThT fluorescence is not corrected, higher Aβ40 concentrations at 2, 5 and 10 µM appear to have a different trend from the lower Aβ40 concentrations at 0.5 and 1 µM. Higher Aβ40 concentrations (2, 5 and 10 µM) show a bell-shaped profile with peak fluorescence at 20–50 µM ThT, while the lower Aβ40 concentrations (0.5 and 1 µM) show a continuous increase in fluorescence intensity over the entire range of ThT concentrations (figure 2*a*). However, when background ThT signal is subtracted, all Aβ40 concentrations give similar bell-shaped profiles, with peak fluorescence at 20 µM ThT for Aβ40 fibril concentrations at 1–10 µM (figure 2*b*).

Here, we propose another way of background correction by presenting the data as fold change in fluorescence intensity over background ThT signal. To calculate fold change, the fluorescence intensity of the protein sample is divided by the fluorescence intensity of the ThT-only sample for each ThT concentration. The fold change profiles (figure 2*c*) are similar to those with background subtraction (figure 2*b*), with peak fluorescence at 20 µM ThT for Aβ40 fibril concentrations at 1–10 µM. In our opinion, fold change is superior to background subtraction because not only does fold change correct the background, it also reports the sensitivity of the measurements in the form of signal-to-background ratio. More importantly, the absolute value of fluorescence intensity is dependent on the type of instrument as well as measurement settings, while the fold change in ThT fluorescence is less dependent on these variables, and can be more easily standardized among different research groups. Therefore, the use of fold change may facilitate direct comparison of aggregation data from different laboratories.

**Figure 3.**
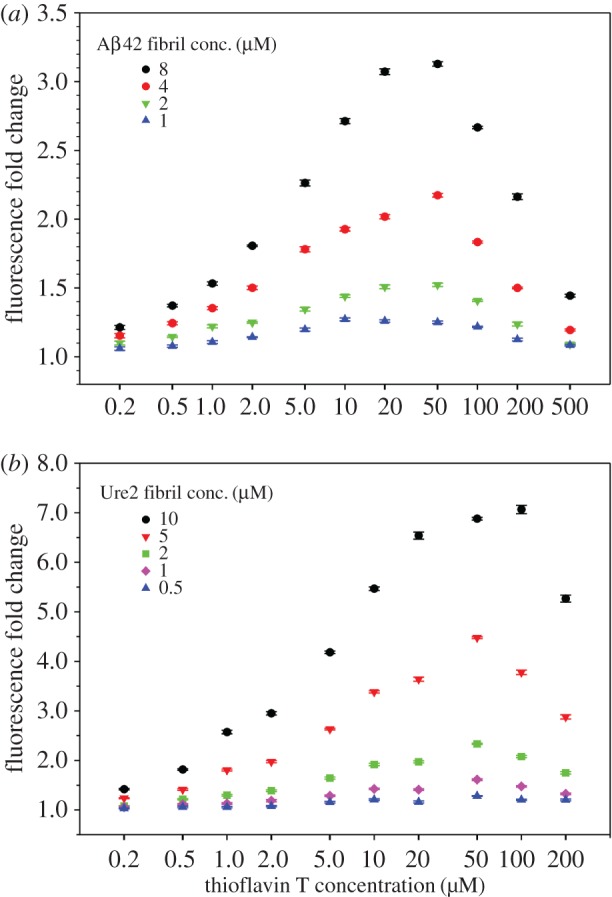
ThT fluorescence at various concentrations of ThT and fibrils of Aβ42 (*a*) and Ure2 (*b*). ThT fluorescence is reported as fold change over ThT background. Error bars are standard deviations of three readings of the same sample and represent instrument measurement fluctuations.

To check whether the maximum fluorescence at 20 µM ThT is a unique feature of Aβ40 fibrils, we performed ThT binding to Aβ42 and yeast prion protein Ure2 fibrils. For Aβ42 fibrils, we studied a combination of 11 concentrations of ThT (0.2–500 µM) and 4 concentrations of Aβ42 (1–8 µM). As shown in figure 3*a*, the same bell-shaped curve is observed with peak fluorescence at 50 µM ThT for most of the Aβ42 fibril concentrations. For Ure2 fibrils, we studied a combination of 10 ThT concentrations (0.2–200 µM) and 5 Ure2 concentrations (0.5–10 µM). As shown in figure 3*b*, fluorescence intensity peaks at 50 µM ThT for most of the Ure2 fibril concentrations. Overall, the ThT binding to Aβ40, Aβ42 and Ure2 fibrils suggest that maximum ThT fluorescence appears at ThT concentrations of 20–50 µM.

Our data also suggest that the maximum ThT fluorescence is not determined by amyloid to ThT ratio. Previously, Younan & Viles [32] studied the binding of ThT to Aβ40 fibrils and concluded that ThT fluorescence depends on amyloid to ThT ratio and a 1 : 1 ratio gives the highest fluorescence signal. In their work, Younan & Viles [32] studied two Aβ40 fibril concentrations: 10 and 20 µM. For 10 µM Aβ40 fibrils, the maximum fluorescence was observed at 10 µM ThT. For 20 µM Aβ40 fibrils, the fluorescence peaked in between 10 and 20 µM ThT, and the fluorescence at 10 µM ThT was very close to the maximum. Therefore, an argument can be made that 10 µM ThT seems to give highest fluorescence signal for both 10 and 20 µM Aβ40 fibrils. In another study of ThT binding with α-synuclein fibrils, Ahn *et al*. [33] showed that, for three α-synuclein fibril concentrations spanning a fivefold range, the ThT concentrations that gave highest fluorescence signals are in a narrow range of approximately 15–30 µM. This is consistent with our findings that the maximum ThT fluorescence mostly depends on the total ThT concentration, rather than amyloid to ThT ratio.

### 2.3. Thioflavin T fluorescence intensity correlates linearly with amyloid fibril concentration

**Figure 4.**
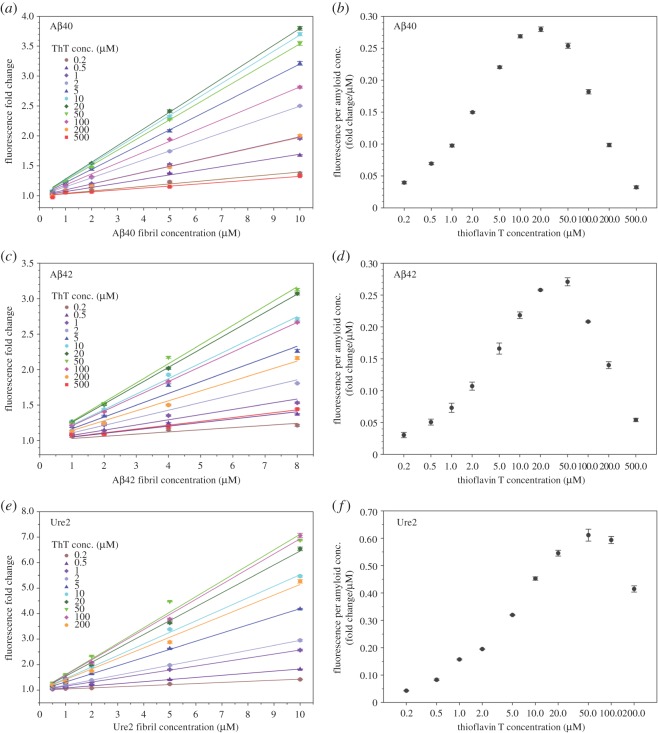
ThT fluorescence correlates linearly with amyloid fibril concentration. (*a,c,e*) ThT fluorescence in the presence of various concentrations of ThT and amyloid fibrils of Aβ40 (*a*), Aβ42 (*c*) and Ure2 (*e*). Lines are the linear regression of the data. The slope of the linear regression is termed ‘FPAC’. The intercept is set at 1.0 for the linear fitting. (*b,d,f*) FPAC is plotted as a function of ThT concentration for Aβ40 (*b*), Aβ42 (*d*) and Ure2 (*f*). Error bars in panels *a*, *c* and *e* are standard deviations of three readings of the same sample and represent instrument measurement fluctuations. Error bars in panels *b*, *d* and *f* are fitting errors from the linear regression.

When looking at ThT fluorescence as a function of amyloid fibril concentration, we find that ThT fluorescence has a linear correlation with the concentration of amyloid fibrils (figure 4). This is the case for all the investigated ThT concentrations between 0.2 and 500 µM and for all three amyloid fibrils investigated here: Aβ40 (figure 4*a*), Aβ42 (figure 4*c*) and Ure2 (figure 4*e*). We fitted the data with a linear regression and the *r*^2^-values are higher than 0.95 for all the fittings (table 1).

**Table 1.** *R*^2^-values from the linear regression of the ThT binding data in figure 4.

	*R*^2^-values from linear regression		
ThT conc. (µM)	Aβ40 fibril data	Aβ42 fibril data	Ure2 fibril data
0.2	0.9887	0.9486	0.9960
0.5	0.9971	0.9750	0.9956
1	0.9978	0.9715	0.9994
2	0.9996	0.9896	0.9999
5	0.9997	0.9918	0.9998
10	0.9996	0.9983	0.9993
20	0.9993	0.9999	0.9985
50	0.9990	0.9983	0.9950
100	0.9986	0.9999	0.9981
200	0.9967	0.9955	0.9970
500	0.9817	0.9929	—

The slope of the linear regression reflects the change in ThT fluorescence as a function of amyloid concentration, and we name this slope as ‘Fluorescence per Amyloid Concentration (FPAC)’. Our data suggest that FPAC is a specific amyloid property at a given ThT concentration. For Aβ40 fibrils, FPAC peaks at 20 µM ThT (figure 4*b*). For both Aβ42 (figure 4*d*) and Ure2 fibrils (figure 4*f*), FPAC peaks at 50 µM. The peaks in the FPAC plots are consistent with the peaks in the ThT fluorescence plot for each individual amyloid concentration (figures 2 and 3).

**Figure 5.**
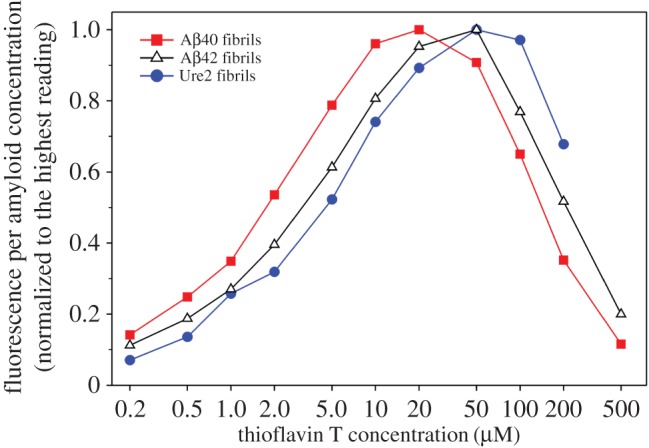
Comparison of the FPAC profile as a function of ThT concentration for Aβ40, Aβ42 and Ure2 fibrils.

We compared FPAC as a function of ThT concentration for Aβ40, Aβ42 and Ure2 fibrils (figure 5). The results show that the peak in these curves shifts to the right for the protein of higher molecular weight. The curve for Aβ40, a 40-residue protein, peaks at the very left, while the curve for Ure2, with an 89-residue prion domain, peaks at the very right. This suggests that the ThT concentration giving maximal FPAC correlates positively with the number of ThT binding sites on the amyloid.

The values of FPAC can be used to quickly estimate the concentration of amyloid for a given sample. In addition, when everything else is equal, different FPAC values may indicate different underlying structures of amyloid fibrils, which may bind to ThT molecules with different affinity or give different quantum yields.

### 2.4. Optimal thioflavin T concentrations for monitoring aggregation kinetics

**Figure 6.**
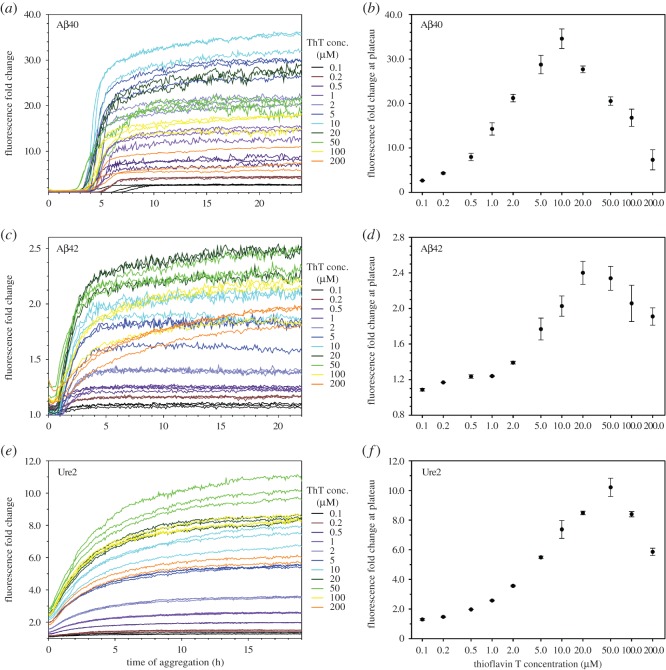
Aggregation kinetics of Aβ40, Aβ42 and Ure2 in the presence of various concentrations of ThT. (*a,c,e*) Kinetics curves in the presence of different ThT concentrations for Aβ40 (*a*), Aβ42 (*c*) and Ure2 (*e*). Aggregation was performed at 37°C without agitation. Protein concentration is 50 µM for Aβ40, and 10 µM for Aβ42 and Ure2. The buffer is PBS for Aβ40 and Aβ42, and PBS containing 0.35 M guanidine hydrochloride for Ure2. (*b,d,f*) Fluorescence at aggregation plateau as a function of ThT concentrations for Aβ40 (*b*), Aβ42 (*d*) and Ure2 (*f*). Symbols represent the average and error bars represent the standard deviation of multiple experiments.

To find out the optimal ThT concentrations for monitoring aggregation kinetics, we performed aggregation kinetics experiments of Aβ40, Aβ42 and Ure2 with a wide range of ThT concentrations (0.1–200 µM). For Aβ40, the aggregation was performed at 50 µM concentration, and the aggregation curves are shown in figure 6*a*. The aggregation data are presented as fold change, which is calculated by dividing the fluorescence intensity of the aggregation sample by the ThT-only sample for each time point of incubation. The fold change of fluorescence intensity at aggregation plateau was plotted as a function of ThT concentration (figure 6*b*). The ThT concentration that gives rise to highest fluorescence at aggregation plateau is 10 µM.

Aβ42 aggregation was performed at 10 µM concentration in the presence of 0.1–200 µM ThT, and the aggregation results are shown in figure 6*c*. The fold change in ThT fluorescence at aggregation plateau is highest at 20 µM ThT (figure 6*d*).

Figure 6*e* shows the results of Ure2 aggregation kinetics at 10 µM, and the fold change in ThT fluorescence versus concentration is shown in figure 6*f*. The maximum ThT fluorescence at aggregation plateau is observed at 50 µM of ThT (figure 6*f*).

Overall, the ThT concentrations giving the maximum fluorescence readings in real-time monitoring of fibril formation are slightly less than (for Aβ40 and Aβ42 fibrils), or similar to (for Ure2 fibrils), those of sonicated fibrils. The shift of peak fluorescence to a lower ThT concentration for real-time aggregation may be due to the bundling of amyloid fibrils, which reduces the number of accessible ThT binding sites.

The results here reinforce our conclusion that the maximal ThT fluorescence mostly depends on total ThT concentration, rather than amyloid to ThT ratio. For Aβ40 aggregation studies, the Aβ40 concentration is 50 µM, and the maximum fluorescence intensity was observed at 10 µM. In this case, the amyloid to ThT ratio is 5 : 1. Notably, as discussed above, the data points of the highest fluorescence in figure 2 corresponds to a wide range of amyloid to ThT ratios (from 1 : 2 to 1 : 20). Yet, across these widely different Aβ40 concentrations, the ThT concentration that gives maximal fluorescence remains in the narrow range of 10–20 µM. Therefore, it is the ThT concentration, rather than the amyloid to ThT ratio, that provides robust predictions on maximal fluorescence intensity.

Our results suggest that ThT concentrations of 10–50 µM provide maximum sensitivity in the studies of aggregation kinetics, although other factors may also affect the selection of ThT concentration. One such factor is the effect of ThT on aggregation kinetics, as discussed later.

### 2.5. The effect of thioflavin T on fibril formation kinetics

**Figure 7.**
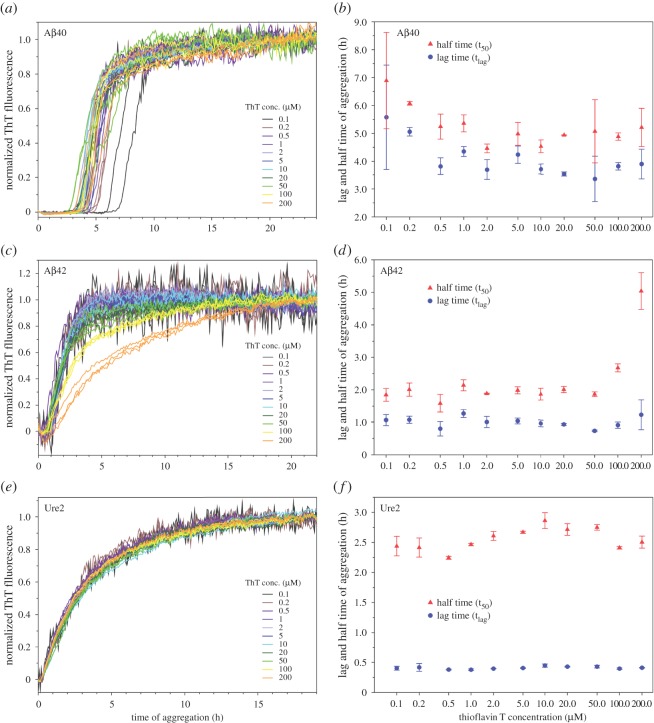
Effect of ThT on aggregation kinetics of Aβ40, Aβ42 and Ure2 proteins. (*a,c,e*) Normalized aggregation curves for Aβ40 (*a*), Aβ42 (*c*) and Ure2 (*e*). (*b,d,f*) Lag time (t_lag_) and half time (t_50_) from the aggregation kinetics for Aβ40 (*b*), Aβ42 (*d*) and Ure2 (*f*). Symbols represent average and error bars represent standard deviation of multiple experiments.

The aggregation experiments in this work allow us to evaluate the effect of ThT on the aggregation kinetics of Aβ40, Aβ42 and Ure2. The rationale is that, if ThT affects aggregation kinetics, we would observe a change in aggregation curves as a function of ThT concentration. To compare the shape of the kinetics curves, we normalize the kinetics traces of different ThT concentrations. Then we determine the lag time (t_lag_), at which ThT fluorescence reaches 5% of the maximum amplitude, and half time (t_50_), at which ThT fluorescence reaches 50% of the maximum amplitude. The results are shown in figure 7.

For Aβ40 aggregation kinetics, all the traces have similar shapes, but different traces are not tightly clustered together (figure 7*a*). The lag time and half time for 0.5 µM and higher ThT concentrations do not show any trend that is related to ThT concentration (figure 7*b*). At very low concentrations of ThT (0.1 and 0.2 µM), the aggregation does appear to be slower with slightly larger t_lag_ and t_50_ values, but the effect is very small. The t_lag_ and t_50_ values for the 0.1 and 0.2 µM ThT curves are only approximately 20% larger than those for the 0.5 µM ThT (figure 7*b*).

For Aβ42 aggregation kinetics, all the traces with ThT concentrations of 20 µM or smaller have a similar shape (figure 7*c*), while traces with 50 µM or higher concentrations of ThT display broader transition regions. The half time for the 100 and 200 µM ThT is much longer than the half time with smaller ThT concentrations, but the half time for the 50 µM ThT remains similar to other ThT concentrations. The effect on lag time is less prominent compared with the half time (figure 7*d*).

For Ure2 aggregation kinetics, all the traces have similar shapes (figure 7*e*) and are clustered tightly together. Lag time and half time (figure 7*f*) do not show any particular ThT concentration-dependent trends, suggesting that ThT has no significant effect on Ure2 aggregation.

**Figure 8.**
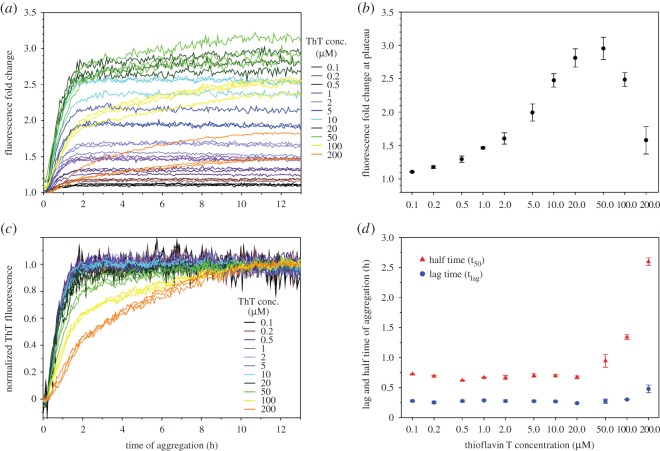
Aggregation kinetics of Aβ42 using a protocol with 0.35 M guanidine hydrochloride. (*a*) Kinetics curves in the presence of different ThT concentrations. Aggregation was performed at 37°C without agitation. Protein concentration is 10 µM. The buffer is PBS containing 0.35 M guanidine hydrochloride. (*b*) Fluorescence at aggregation plateau as a function of ThT concentrations. Symbols represent the average and error bars represent the standard deviation of three experiments. (*c*) Normalized aggregation curves. (*d*) Lag time (t_lag_) and half time (t_50_) from the aggregation kinetics. Symbols represent average and error bars represent standard deviation of three experiments.

For the results shown in figure 7, PBS buffer was used for the aggregation experiments of Aβ40 and Aβ42. However, PBS buffer containing 0.35 M guanidine hydrochloride was used for Ure2 aggregation. This is due to the high aggregation propensity of Ure2. To obtain reproducible kinetics data, it is necessary to prepare Ure2 stock solutions in 7 M guanidine hydrochloride and to start aggregation by diluting the Ure2 stock solution into PBS buffer. There is a question about how the presence of 0.35 M guanidine hydrochloride affects aggregation kinetics. To address this question, we performed Aβ42 aggregation using a protocol similar to Ure2 aggregation and the results are shown in figure 8. The overall shape of the kinetics curves with 0.35 M guanidine hydrochloride in figure 8*a* is very similar to the kinetics curves in just PBS buffer in figure 6*c*. The fluorescence peaks at 50 µM ThT with 0.35 M guanidine hydrochloride (figure 8*b*), compared to 20 µM ThT in just PBS (figure 6*d*). The fold change in fluorescence at aggregation plateau is also very similar: 2.8 at 20 µM ThT for the aggregation with 0.35 M guanidine hydrochloride (figure 8*b*), 2.4 at 20 µM ThT in PBS buffer (figure 6*d*). The normalized kinetics traces for ThT concentrations of 20 µM or lower are all superimposed on one another (figure 8*c*). For ThT concentrations of 50 µM or higher, changes in kinetics curves and aggregation parameters are observed (figure 8*c,d*). This is also similar to the results using just PBS buffer. Therefore, these results suggest that the kinetics data in the presence of 0.35 M guanidine hydrochloride are extremely similar to those obtained with just PBS buffer.

Overall, our data suggest that ThT has little effect on aggregation at low concentrations (20 µM or lower). For Aβ40, slightly larger values of t_lag_ and t_50_ for 0.1 and 0.2 µM ThT suggest that ThT may promote Aβ40 aggregation, but again, the effect is very small. At high concentrations (50 µM and higher), ThT changes the shapes of the aggregation curves for Aβ42, but not for Aβ40 and Ure2. These results suggest that the effect of high ThT concentration on aggregation process is protein-dependent.

## 3. Material and methods

### 3.1. Preparation of Aβ40, Aβ42 and Ure2

Aβ was expressed in *Escherichia coli* as a fusion protein, GroES-ubiquitin-Aβ, and then cleaved with a deubiquitylating enzyme to obtain full-length Aβ without any extra residues. The expression and purification of Aβ40 and Aβ42 were performed as previously described [25,34,35]. The DNA constructs of wild-type GroES-ubiquitin-Aβ [36] and the deubiquitylating enzyme Usp2cc [37] were kindly provided by Dr Il-Seon Park at Chosun University (South Korea) and Dr Rohan T. Baker at Australian National University (Australia). The prion domain of yeast prion protein Ure2 was expressed as a fusion protein with the M-domain of Sup35 at the C-terminal end of Ure2, and was used without removing the Sup35-M domain. The expression and purification of Ure2 was performed as previously described [26,38].

### 3.2. Preparation of sonicated amyloid fibrils

Aβ40 and Aβ42 proteins were treated with HFIP before use. We first dissolved Aβ40 and Aβ42 powder in HFIP to a protein concentration of 500 µM. The samples in HFIP were left on the bench for 30 min, and then HFIP was evaporated in a chemical hood overnight. Finally, the samples were put under vacuum for 1 h to complete HFIP treatment. To make amyloid fibrils, HFIP-treated Aβ40 and Aβ42 proteins were dissolved in CG buffer (20 mM CAPS, 7 M guanidine hydrochloride, pH 11), and concentration was determined using absorbance at 280 nm and an extinction coefficient of 1.28 mM^−1^ cm^−1^ [39]. After concentration measurements, Aβ40 and Aβ42 samples were diluted 20-fold into PBS buffer (50 mM phosphate, 140 mM NaCl, pH 7.4), and then incubated at 37°C for 5 days to allow fibril formation. The progress of aggregation was monitored using thioflavin T. The final concentration of Aβ40 in the aggregation reaction is 40 µM, and the final concentration of Aβ42 in the aggregation reaction is 15 µM. The fibril concentrations were considered to be the same as the starting monomer concentration, with the assumption of complete conversion from monomers to fibrils.

Ure2 protein powder was first dissolved in PG buffer (15 mM sodium phosphate, 7 M guanidine hydrochloride, pH 6.8) to a concentration of 400 µM. Then the Ure2 sample was diluted 20-fold into PBS buffer and was incubated at 37°C for 5 days. The Ure2 fibril concentration was considered to be 20 µM, assuming complete conversion from monomers to fibrils.

Before ThT binding, the fibrils of Aβ40, Aβ42 and Ure2 were sonicated for 200 s using a Branson Digital Sonifier model 450 (microtip, 10% amplitude) with intermittent pause to allow samples to cool down.

### 3.3. Thioflavin T preparation and fluorescence measurement

ThT (Sigma, product no. T3516) was dissolved in PBS buffer and was filtered through a 0.2 µm syringe filter. Then the concentration of thioflavin T was determined using an extinction coefficient of 36 mM^−1^ cm^−1^ at 412 nm [27,28].

For measurement of ThT fluorescence without proteins, various concentrations of ThT were prepared in PBS using serial dilution. Forty microlitres of ThT sample at each concentration was transferred to a black 384-well Nonbinding Surface microplate with clear bottom (Corning product no. 3655). The ThT fluorescence was measured at room temperature (approx. 24°C) using a Victor 3 V plate reader (Perkin Elmer) through the bottom of the plate with excitation filter of 450 nm and emission filter of 490 nm.

For measurement of ThT fluorescence in the presence of amyloid fibrils, various concentrations of sonicated Aβ40, Aβ42 and Ure2 fibrils were also prepared using serial dilution. Then 20 µl of ThT was mixed with 20 µl of sonicated fibrils to achieve desired concentrations of ThT and amyloid. Fluorescence measurements were performed as described above.

### 3.4. Fibrillization kinetics

For Aβ40, HFIP-treated powder was dissolved in 10 mM NaOH. Then eight volumes of PBS buffer and one volume of 10 mM HCl solution were added, in that order. Aβ40 sample was mixed with various concentrations of ThT and then transferred to microplate for kinetics studies. The final concentration of Aβ40 was 50 µM.

For Aβ42, HFIP-treated powder was dissolved in CG buffer, and was then buffer exchanged to PBS using a 5 ml HiTrap desalting column (GE healthcare). The concentration of Aβ42 was determined using fluorescamine [40], and hen egg white lysozyme was used to prepare the standard curve. Aβ42 was then diluted to 10 µM in PBS buffer containing various concentrations of ThT. In an alternative protocol, HFIP-treated Aβ42 was dissolved in CG buffer to 200 µM concentration, and was then diluted 20-fold to PBS buffer containing various concentrations of ThT. The final Aβ42 concentration was also 10 µM.

Ure2 protein was dissolved in PG buffer to 200 µM concentration, and was then diluted 20-fold to PBS buffer containing various concentrations of ThT. The final concentration of Ure2 was 10 µM.

The volume of aggregation was 50 µl for all the samples. Samples were kept on ice whenever possible. All samples were transferred to a black 384-well Nonbinding Surface microplate with clear bottom (Corning product# 3655), and sealed with a polyester-based sealing film (Corning product# PCR-SP). To begin aggregation, the microplate was transferred to a Victor 3 V plate reader (Perkin Elmer) and was incubated at 37°C without agitation. The ThT fluorescence was measured through the bottom of the plate approximately every 5 min, with excitation filter of 450 nm and emission filter of 490 nm.

## Data accessibility

Data available from the Dryad Digital Repository: http://dx.doi.org/10.5061/dryad.b8c4r [41].
